# Role of Natural Radiosensitizers and Cancer Cell Radioresistance: An Update

**DOI:** 10.1155/2016/6146595

**Published:** 2016-02-22

**Authors:** Arif Malik, Misbah Sultana, Aamer Qazi, Mahmood Husain Qazi, Gulshan Parveen, Sulayman Waquar, Abdul Basit Ashraf, Mahmood Rasool

**Affiliations:** ^1^Institute of Molecular Biology and Biotechnology (IMBB), The University of Lahore, Pakistan; ^2^Center for Research in Molecular Medicine (CRiMM), The University of Lahore, Pakistan; ^3^University College of Medicine and Dentistry, The University of Lahore, Pakistan; ^4^Center of Excellence in Genomic Medicine Research (CEGMR), King Abdulaziz University, Jeddah, Saudi Arabia

## Abstract

Cancer originates from genetic mutations accumulation. Cancer stem cells have been depicted as tumorigenic cells that can differentiate and self-renew. Cancer stem cells are thought to be resistant to conventional therapy like chemotherapy and radiation therapy. Radiation therapy and chemotherapy damage carcinomic DNA cells. Because of the ability of cancer stem cells to self-renew and reproduce malignant tumors, they are the subject of intensive research. In this review, CSCs radioresistant mechanisms which include DNA damage response and natural radiosensitizers have been summed up. Reactive oxygen species play an important role in different physiological processes. ROS scavenging is responsible for regulation of reactive oxygen species generation. A researcher has proved that microRNAs regulate tumor radiation resistance. Ionizing radiation does not kill the cancer cells; rather, IR just slows down the signs and symptoms. Ionizing radiation damages DNA directly/indirectly. IR is given mostly in combination with other chemo/radiotherapies. We briefly described here the behavior of cancer stem cells and radioresistance therapies in cancer treatment. To overcome radioresistance in treatment of cancer, strategies like fractionation modification, treatment in combination, inflammation modification, and overcoming hypoxic tumor have been practiced. Natural radiosensitizers, for example, curcumin, genistein, and quercetin, are more beneficial than synthetic compounds.

## 1. Introduction

Cancer is now the most common cause of human death. Although many advances have been made in the treatment of cancer, still mortality rate of cancer malignancies remains the same, due to the tumor resistance to radiation therapy. The most common cause of failure is the development of secondary tumors and metastasis. Cancer is widely characterized by the abnormal growth of cells having genetic/epigenetic changes, which results in high rate of morbidity and mortality. With the declaration of cancer war, substantial steps are made in the battle of cancer biology for more understanding. Thus, antitumor therapy showed evident results against the genetic material of tumor destruction. Usually, the carcinomic DNA cells are damaged during radiation and chemotherapy. Commonly, cancer treatment includes both radiation and chemotherapy depending on the patient condition and wound type. Still, cancer is found to be one of the incurable diseases. The inadequate prognosis and late detection of malignancy made the survival rate lower.

Oncologists face another problem during treatment when renewal of tumor takes place. Metastasis happens by evading the DNA-damaged induced cells and makes the tumor cells able to regrow; thus, they are termed cancer stem cells. Although the mechanisms are still not clear, worldwide work is going on to illuminate CSC's resistance. Cancer stem cells due to their ability to self-renew and reproduce malignant tumors are the subject of intensive research. Stem cells by asymmetric cell division give rise to progenitor cell into differentiated cells. Cancer stem cells as compared with progenitor cells have very low rate of development [1]. Normally, origin of cancer occurs after genetic and epigenetic changes within the cell as a result abrogating genomic instability. It has been suggested that hierarchical original tissue structure is preserved by abnormal clones, at the start of malignancy. Through model of cancer stem cell, it is proposed that differentiated cells are produced by transit-amplifying cells [2].

## 2. Cancer Stem Cells and Radiation Therapy Response

Radiation therapy provides cure in many types of tumors. Radiotherapy in the initial stage of tumor procession can control tumor [3]. Radiation therapy is one of the effective treatments for glioblastoma, but still tumor renewal causes death of the patient. It was analyzed that cancer stem cells survive more during radiotherapy than noncancer stem cells. Although radiotherapy damages the same amount of DNA in both cancer stem cells and noncancer stem cells, cancer stem cells robustness ability to repair damage was dominant. After radiotherapy, noncancer stem cells showed more apoptosis. Genotoxic stress which is produced due to the damage to organism genome triggers ATM, CHK1, and CHK2 (serine/threonine-protein kinase) proteins which further triggers DNA repair pathway. Cancer stem cells exhibit basic level of checkpoint activation and are always ready against genotoxic stress. Using CHK1 and CHK2 inhibitors helps in radiation sensitivity of cancer stem cells [4]. During radioresistance, Wnt/*β*-catenin plays an important role. Radiation improves stem cells of murine mammary epithelial cell line that had antiapoptotic proteins, that is, activated *β*-catenin and survivin. These cells have the ability to self-renew elevation in mammosphere formation assay [5, 6]. Radiotherapy results in low reactive oxygen species (ROS) in breast cancer stem cells as compared to noncancer stem cells. This decrease in ROS level of breast could be the result of raised radical scavenger properties.

## 3. Reactive Oxygen Species (ROS)

Reactive oxygen species have a very important role in the physiological developmental processes, for example, emergence of embryonic stem cells/differentiation of embryonic cardiomyocytes [7–9]. It has been demonstrated that reactive oxygen species are concerned with many biological processes like expression of genes, translational proteins, and protein-protein interactions [10]. ROS function in cellular signaling, signals propagation, and balancing of cellular input. They function as variable resistor to organize several cellular processes and set the cellular activity [11]. With the growing advancement in genomics and proteomics, many pathways give information about the balancing of reactive oxygen species and how cellular processes are controlled. Particularly in stem cells, change in state of oxidation, also called redox regulation, may be responsible for communicating mitochondria and nucleus [12–14]. Redox-mediated communication between mitochondria and nucleus explains the cellular metabolism coordination with remodulation of chromatin, cell cycling, expression of gene, repairing of DNA, and cell differentiation. Reactive oxygen species are also concerned with the process of ageing but much is not known about the involvement of ROS in stem cells ageing [15, 16].

## 4. Genesis and Scavenging of Reactive Oxygen Species

**Figure 1:**
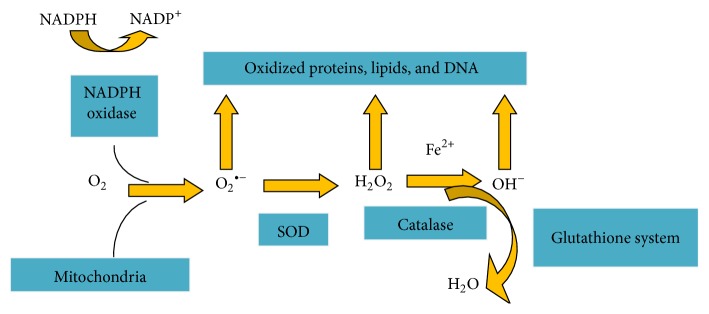
ROS have superoxide anion, hydrogen peroxide, and hydroxyl radical. Superoxide anion (O_2_^∙−^) generated from NADPH oxidation through NADPH oxidases. It reduces to hydrogen peroxide (H_2_O_2_) where superoxide dismutase (SOD) acts as catalyst. Hydrogen peroxide further reduces to H_2_O via catalase/oxidized iron (Fe^2+^) to highly reactive hydroxyl ion (OH^−^). During oxidative stress, when generation of reactive oxygen species spaces out their scavenging system, levels of oxidized reactive oxygen species accumulate and this damages many cellular factors.

Reactive oxygen species originate from molecular oxygen reducing one electron (Figure 1). Mainly intracellular ROS are of three kinds: hydrogen peroxide (H_2_O_2_), superoxide anion (O_2_^−^), and hydroxyl radical (OH^−^). Superoxide anion comprises unpaired electron that rapidly reacts and reduces to hydrogen peroxide through superoxide dismutase (SOD), an antioxidant enzyme [17]. Hydrogen peroxide further reduces to water (H_2_O) and oxide ion (O_2_) through several cellular antioxidants (Figure 1). Reactive oxygen species can be observed intracellularly through many techniques, but still many tries have failed to discriminate within different ROS species. Hydrogen peroxide (H_2_O_2_) is considered as the major ROS species in signaling of intracellular molecules and in particular circumstances can directly play a role as second messenger and in integration of environmental cues and finally pass them to signal transduction cascade down. This is usually due to longer half-life of hydrogen peroxide (H_2_O_2_) and ability to discriminate via membranes comparatively with other kinds of reactive oxygen species [10].

During normal physiological conditions, ROS scavenging regulates the reactive oxygen species generation. ROS scavenging system includes such antioxidant enzymes that have the ability to directly neutralize reactive oxygen species and electron acceptance from reactive oxygen species. The abnormal production of ROS leads to OS (oxidative stress) which affects adversely multiple cellular components, that is, proteins, lipids, and DNA. Within the cell, specific antioxidants are specific for ROS species that prevents pathological levels of reactive oxygen species and repairs the cellular oxidative damage. Superoxide dismutase (SOD), catalase, peroxiredoxins (PRX), thioredoxin (TRX), glutathione peroxidase (GPX), and glutathione reductase (GRX) are included. Among all of these antioxidants, glutathione (a tripeptide) is mostly synthesized by the cell. Glutaredoxin and thioredoxin reduce the oxidized proteins and hydrogen peroxides while superoxides and catalases reduce O_2_^−^ and H_2_O_2_. The other subcellular antioxidants present at increased generation of ROS, like mitochondria, raise the ROS scavenging efficiency.

## 5. Reactive Oxygen Species in Metabolism of Stem Cell

The process of catabolism and anabolism is termed cellular metabolism in which chemical carbon converts substrates for energy generation in the form of ATP. The reduction of cofactors is called catabolism and production of macromolecules precursors like lipids, nucleotides, and amino acids is known as anabolism. The cellular processes can shift the balance of catabolic and anabolic processes. The cellular processes may include growth and proliferation which mostly yield building blocks of deoxyribonucleic acids (DNA), proteins, and membranes through anabolism. Metabolic pathways can directly affect stem cells by remaining inactive, self-renew, or differentiate [18–20]. The changes in ROS levels can affect signaling pathways. The regulation of cell-cycle progression, apoptosis, and quiescence/differentiation can be altered through ROS by reacting with proteins like kinases and phosphatases/transcription factor [21–23]. The metabolic enzymes/proteins that direct the metabolic flux in nutrient-sensing pathways can be modified directly by ROS [24–26]. As a result, reactive oxygen species can be the signaling molecules that can play a role in both metabolism and stem cell. Significantly, cell membrane can be affected via different ROS-independent mechanisms. Mechanisms like epigenesis and functioning of metabolic enzymes can be changed [27–31]. Still, comparing the ROS effects, the discussions on metabolism and cell membrane methods are not considerably characterized in the stem cells.

## 6. Damaged DNA Response/DNA Signaling

Radiation-induced cell death may take place through direct/indirect transfer of energy to cellular structures that include plasma membrane, mitochondria, and chromatin. During replication stress or DSBs, proteins/enzymes like Ataxia Telangiectasia Mutated (ATM), Ataxia Telangiectasia/Rad3-related kinase (ATR), and CHK1 and CHK2 also get engaged. These mechanisms of cell-cycle arrest allow the enlistment of repair failure and irreversible damage of proapoptotic molecules [32]. The common observation for cancer stem cells resistance to radiation therapy is believed to occur due to their DNA damage repairing ability that is provoked by radiation/chemical drugs. This repairing of DNA damage can be by elevation of DNA repair mechanism directly or by cell-cycle progression indirectly. During DDR, in normal and malignant cells, one of the important DNA DSBs sensors is called MRN complex, that is, proteins like MRE11, RAD50, and NBS. MRN complex has a major role in binding and stabilization of broken DNA ends and also activates ATM. The functioning of MRN complex by BMI is interlinked with cancer stem cell molecules like Notch1, ALDH1A1, CD44, and Sonic Hedgehog, along with telomere biology, deregulation, tumor behavior, and prognosis of disease [33].

It has been observed that ATM kinase, the important signaling effector of DDR, plays a major role in DNA damage resistance of cancer stem cells. ATM contains an important sensor of DNA damage and kinase effector downstream that plays the major role in cell-cycle control regulation, DNA repair, senescence development, and apoptosis. It has been indicated that ATM also plays its role in maintenance and proliferation of normal stem cells. ATM plays two major roles: a role in the survival of stem cell and significantly in DDR part, in stem cell maintenance pathway [34, 35]. By considering the first role, ATM is concerned for the survival of neuronal stem cells (NSCs). According to the exact mechanism, although the expression of ATM in neuronal stem cells is abundant, during cell differentiation, it reduces gradually. This hypothesis indicates that for NSC functioning and survival ATM is very important [36]. For maintenance of normal self-renewal and proliferation of neuronal stem cells, ATM is involved by controlling redox status. NSCs become defective for proliferation with ATM loss through oxidative-stress-dependent p38 MAPK signaling, which suggests that p38 is the main key in the ATM/NSCs defective proliferation caused by oxidative stress [37, 38]. Furthermore, it has been depicted that ATM by its function in DDR plays the key role in human neural stem cell terminal differentiation [39]. Moreover, for maintenance of stem cell, ATM protein plays the major role in signaling pathways. Further, ITCH E3-ubiquitin ligases activity is regulated by ATM. ITCH belongs to the family of NEDD4, which is the protein family taking part in different signaling pathways like TNF*α*, DNA damage response, Notch, and Sonic Hedgehog [40].

## 7. miRNAs CSC Resistance

MicroRNAs are a type of endogenous noncoding RNAs. Modifications during regulation of cancer stem cells against genotoxic insults include gene expression, which is regulated by microRNAs (miRNAs). Through the invention of miRNAs, many new ways have been opened in the world of science about gene expression regulation and functioning of various cells, like differentiation, apoptosis, therapy resistance, and proliferation [41]. For many years, it has been proved that cancer development is linked with these tiny genetic regulators. The miRNAs indicated huge information regarding history and tumors state differentiation and thus molecular link has been provided between cancer stem cells and normal stem cells. The expression of miRNAs might have adverse results for functioning of cancer cells for tumor radiation resistance. Yan and coworkers were the first to present the notion that DNA repair machinery could be targeted by miRNAs and thus tumor cells sensitized to radiation [42]. Now researchers proved that miRNAs can regulate tumor radiation resistance [43–46].

## 8. Radiotherapy

Radiation therapy is the most effective tool against treatment of cancer. High doses of radiation are used in radiation therapy to halt growth of tumor. Ionizing radiation (IR), like X-rays and gamma-rays, is commonly used for the treatment of cancer because it has the ability to pass through tissues and can break chemical bonds and help in the removal of electrons from atoms to get ionized. The ionized ions as a result damage cancer cells. Cancer cells are not killed immediately by ionizing radiation; in fact, substantial time is required for killing of cancer cells. Ionizing radiation can decrease the signs and symptoms induced by a growing tumor. To increase the effect of therapies, ionizing radiation is often given before, during, and even after the surgery. The exposure of ionizing radiation can be external and internal. External beam radiation like X-rays or *γ*-rays targets the particular part of the cancerous patient. Therapies of internal radiation have neutrons, electrons, protons, *α* or *β* particles, and carbon ions in which solid or liquid radiation is placed within the body. The use of ionizing radiation to kill cancer cells biologically depends on the kind of radiation being given, amount of dosage, rate of fractionation, and the organ to be targeted [47].

Although radiotherapy is one of the most effective treatments for cancer, still a large number of patients had radioresistance of their cancers. Ionizing radiation if given alone is found to be more effective against few cancers like non-small-cell lung cancer, cervical cancer, larynx cancer, skin cancer, head and neck cancer, prostate cancer, and lymphomas, but it is not found to be effective for the cancers like breast cancer, glioblastoma, advanced non-small-cell lung cancer, bladder cancer, and soft tissue carcinoma, maybe because of intrinsic radioresistance of cancer cells [48]. Ionizing radiation combined with other modes of treatment gives hope against radioresistant cancers. Ionizing radiation can cause DNA damage directly or indirectly. Basically, the radiation-induced bystander effect is the major part of ionizing radiation mediated damage which transfers the irradiated damage signals to unirradiated cells of cancer. Another hypothesis is that bystander effect also plays its role in imbalance of genome and carcinogenesis [49]. The most important constituents of IR-induced bystander effect are reactive oxygen species [50, 51]. This phenomenon also involves cytokines, activated macrophages, and nitric oxide (NO) [52].

Cellular senescence by permanently arresting the cells growth maintains a substantial tumor-suppressive effect and also damages the surrounding microenvironment. Among cellular senescence, the senescence-associated secretory phenotype (SASP) causes deleterious effects, promotes the proinflammatory responses, and results in progression of tumor [53]. Senescent cells activate the factors of senescence-associated secretory phenotype, that is, soluble signaling and proteases, to maintain potential effects. The major factors of SASP are interleukins, angiogenic factors, inflammatory cytokines, and growth and also have an impact on the neighboring cells. Senescent cells also release proteases like matrix metalloproteinases (MMPs) which contributes to remodeling of tissue [54]. Further, cells which undergo senescence enhance expression of fibronectin [55]. Different biological processes are maintained by fibronectin, that is, adhesion of cell, growth, migration, survival, and differentiation. Moreover, other than proteins, senescent cells can secrete molecules that can maintain tissue microenvironment. Collectively, all of the SASPs which are released by senescent cells have the ability to modify microenvironment by changing the receptors of cell-surface signal transduction. SASP can cause reinforcement of senescence in damaged cells. It has been suggested that IR-induced damage and premature senescence can be developed by the rise in secretory inflammatory signals [56]. Thus, IR-DNA damage activates secretory program. The activation of secretory program determines the radioresistance tumor response by affecting the microenvironment and interaction with other cells of tumor.

## 9. Overcoming Resistance Schemes to Ionizing Radiation

**Figure 2:**
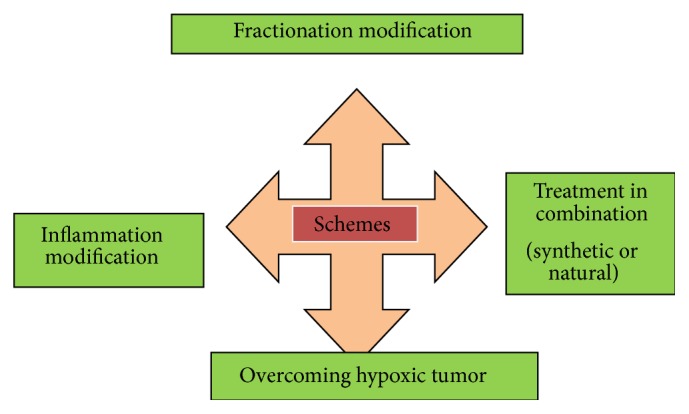
Schemes in treatment of cancer to overcome radioresistance. This includes fractionation modification, treatment in combination, inflammation modification, and overcoming hypoxic tumor.

Radiation-induced programmed cell death is one of the major forms of death in tumors which are derived from lymphoid, hematopoietic, and germ cells. Still, epithelial solid tumors show wide resistance to apoptosis induced by ionizing radiation. Radioresistance is a serious concern, inducing failure of radiotherapy and consequent tumor relapse. Thus, new therapeutic radiosensitizers are desperately required to overcome tumor radioresistance and to improve radiotherapy outcome. Inhibitors which have the ability to target particular constituent of radioresistance pathways are developed for clinical purpose. Further, to compensate synthetic inhibitors limitations, natural radiosensitizers have been formulated. Various strategies have been proposed in the treatment of cancer to overcome resistance to ionizing radiation (Figure 2).

## 10. Fractionation Modification

Radiation fractionated therapy for treatment of cancer has many advantages over single radiation administration as it raises the effect of anticancer therapy and decreases the chances of side effects occurrence in normal tissues. Formally, a total of 2.0 Gy per day, 10 Gy per week, with almost 60 Gy of radiation, is given for six weeks. Unluckily, this scheme cannot control locally elevated cancers sufficiently and because of efficacy limitation and side effects occurrence is not acceptable to patients. Hypofractionation, a novel strategy, has been advised to compensate fractionated radiation therapy limitations. At the beginning, clinical trials are to evaluate the administration per fraction of larger doses in fewer fractions of radiotherapy. Thus, next, hypofractionated radiation therapy will be largely in use compared with conventional strategy, as hypofractionation gives potent results; radiation beams are more focused to tumors. Hypofractionated radiation therapy has higher single dose of fraction but comparing with conventional radiation therapy total dose given is lower. The treatment of hypofractionation radiotherapy is expected to be beneficial in future use. Still researches are being made to investigate more ways of external beam radiation therapy which involves hyperfractionation and also hypofractionation. Hypofractionation treatment has shorter duration as compared with conventional treatment. Hypofractionated radiotherapy is very advantageous, especially for tumors growing rapidly.

## 11. Treatment in Combination

To defeat tumor radioresistance, researchers are much focused to develop tumor-specific radiosensitizers. For clinical practice, combined anticancer therapy is found to be more effective. For treating solid tumors, chemotherapy and radiotherapy in combination have more good results rather than treating with single therapy. The basic principle of this is that single agents of chemotherapy or radiotherapy have lower activity while combined agents show synergistic, anticancer effects.

## 12. Synthetic Targets

So, to target clinically developed DNA DSB repair pathways, many radiosensitizing agents have been formulated. Since the entire HR much correlate with radioresistance, searching ways to inhibit HR repair pathway might be beneficial in cancer cells. For inhibition of HR, many radiosensitizers have been discovered, for example, nucleoside and base analogs like gimeracil, gemcitabine, pentoxifylline, TAS-106, and caffeine [57]. Cox-2 inhibition, the most fundamental enzyme of inflammatory response, uses pharmacological inhibitors like celecoxib, SC-236, and coxibs, to act as radiosensitizers [58]. The HDAC inhibitor PCI-24781, the HSP90 inhibitor 17-allylamino-17-demethoxygeldanamycin, the tyrosine kinase inhibitors imatinib and erlotinib, and the proteasome inhibitor MG132 all target HR repair pathway to radiosensitize cancer cells [57]. Radiotherapy shows novel role as therapeutic partner of cancer immunotherapy. Ionizing radiation plays its role as immune adjuvant, leading to systemic antitumor immunity. IR activates various immunological proteins and transcription factors that regulate immune mediators expression that might elevate development of cancer. Therefore, targeting ionizing radiation-induced inflammatory signaling pathways improves radiotherapy by increasing radiosensitivity. During the treatment, radiotherapy and cisplatin in combination may redistribute calreticulin (a cisplatin-binding protein) by complementing the intrinsic inability of cisplatin drug and thus affect the cell death. The combined treatment of radiotherapy and poly(ADP-ribose), which is the polymerase inhibitor veliparib, through the elevation of tumor immunogenicity maintains its effect [59]. Many studies have proved that radiation therapy and immune stimulation in combination stimulate antitumor immunity, increasing cell death.

## 13. Natural Radiosensitizers

**Figure 3:**
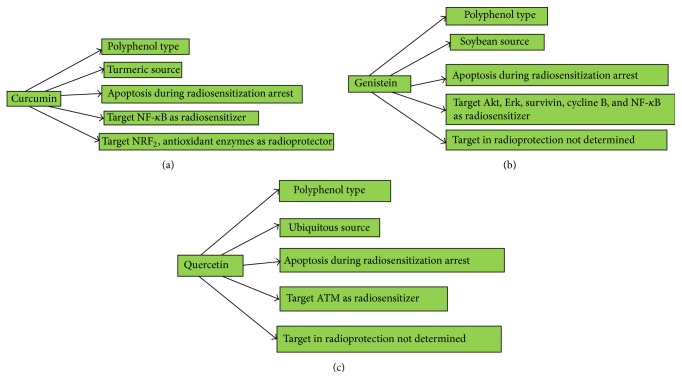
(a) Curcumin is a type of polyphenol. It has many anticancer activities. The source of curcumin is turmeric. It targets NF-*κ*B as radiosensitizers. Curcumin has radioprotective effect and targets Nrf_2_. (b) Genistein, a natural radiosensitizer, is a type of polyphenol. Genistein is a derivative of soybean. It has the ability to inhibit the growth of cancer cell through apoptosis. It can elevate the efficacy of radiation therapy by combining with ionizing radiation. Genistein suppresses Akt and Erk, reduces survivin and cyclin B expression in cervical cancer, and inhibits NF-*κ*B. It acts as radioprotector but the target is not yet determined. (c) Quercetin is the main flavonoid component. It can behave as antioxidant, anti-inflammatory, and antiviral. Quercetin causes apoptosis and arrests the cell cycle. Quercetin by inhibiting ATM elevates radiosensitization. It has radioprotective effect against radiation therapy.

The synthetic inhibitors show limited improvement in treatment and also have more side effects. For these limitations, more and new radiosensitizers are needed to be developed. Radiosensitizers found naturally in foods are believed to be safer than synthetic compounds. Furthermore, natural products because of antioxidant and immune-enhancing effects have improved effects as biological and radiation protectors for normal cells. Few natural radiation sensitizers are curcumin, genistein, and quercetin. Their effect and action as radioprotector in cancer treatment are shown in Figures 3(a), 3(b), and 3(c).

## 14. Inflammation Modification

Recently, to enhance efficacy of radiation therapy, oncologists are paying attention to tumor stroma. Reducing tumor-associated macrophages increases IR antitumor effects; for example, VEGF-neutralizing antibodies in macrophages through IR-induced VEGF downregulation increase antitumor response to ionizing radiation [60]. Immune strategy along with local radiation maintains synergistic antitumor activity. As radiation therapy raises in situ vaccination, few tries have tested radiation therapy and cancer vaccines in combination [61].

## 15. Overcoming Hypoxic Tumor

Tumor hypoxia is believed to be the major problem for radiotherapy as tumor cells of hypoxia are likely to survive ionizing radiation. Various strategies have been introduced against tumor hypoxia. Methods commonly used against tumor hypoxia-related resistance are radiation fractionation, high linear energy transfer (LET), and bioreductive drugs implementation.

## 16. Conclusions

Cancer is the leading cause of death in the world among different diseases. Radiation therapy is beneficial for those patients in whom surgery cannot be done by shrinking or damaging tumor. Tumor exposure to ionizing radiation activates changes, from biochemical changes to various forms of death. The number of doses and fractionation of ionizing radiation determine the level of cellular damage. Anticancer activity maintained by IR is through DNA lesions, like double strand breaks (DSBs), single strand breaks (SSBs), and DNA and base modifications. As radiation is effective for some tumors, other types of tumors are resistant to conventional radiotherapy like glioblastoma multiforme and pancreatic carcinoma, which creates difficulty in targeting. Radiosensitizers are urgently needed to elevate the radiotherapy effects and defeat tumor radioresistance. During treatment, chemotherapy and radiation therapy in combination were found to be successful for various types of tumors. Radiosensitization also targeted DNA damage response. Various trials made with drugs to destroy cancer cells have been found to be effective after radiation but radiosensitizers drugs have shown disappointing results. It has been concluded that novel ways and strategies are still needed to overcome radioresistance in cancer treatment.

## References

[1] Reiman J. M., Knutson K. L., Radisky D. C. (2010). Immune promotion of epithelial-mesenchymal transition and generation of breast cancer stem cells. *Cancer Research*.

[2] Valent P., Bonnet D., De Maria R. (2012). Cancer stem cell definitions and terminology: the devil is in the details. *Nature Reviews Cancer*.

[3] Krause M., Yaromina A., Eicheler W., Koch U., Baumann M. (2011). Cancer stem cells: targets and potential biomarkers for radiotherapy. *Clinical Cancer Research*.

[4] Bao S., Wu Q., McLendon R. E. (2006). Glioma stem cells promote radioresistance by preferential activation of the DNA damage response. *Nature*.

[5] Chen M. S., Woodward W. A., Behbod F. (2007). Wnt/*β*-catenin mediates radiation resistance of Sca1^+^ progenitors in an immortalized mammary gland cell line. *Journal of Cell Science*.

[6] Woodward W. A., Chen M. S., Behbod F., Alfaro M. P., Buchholz T. A., Rosen J. M. (2007). WNT/*β*-catenin mediates radiation resistance of mouse mammary progenitor cells. *Proceedings of the National Academy of Sciences of the United States of America*.

[7] Harris J. M., Esain V., Frechette G. M. (2013). Glucose metabolism impacts the spatiotemporal onset and magnitude of HSC induction in vivo. *Blood*.

[8] Hernández-García D., Wood C. D., Castro-Obregón S., Covarrubias L. (2010). Reactive oxygen species: a radical role in development?. *Free Radical Biology and Medicine*.

[9] Hom J. R., Quintanilla R. A., Hoffman D. L. (2011). The permeability transition pore controls cardiac mitochondrial maturation and myocyte differentiation. *Developmental Cell*.

[10] Holmström K. M., Finkel T. (2014). Cellular mechanisms and physiological consequences of redox-dependent signalling. *Nature Reviews Molecular Cell Biology*.

[11] Liang R., Ghaffari S. (2014). Stem cells, redox signaling, and stem cell aging. *Antioxidants and Redox Signaling*.

[12] Gomes A. P., Price N. L., Ling A. J. Y. (2013). Declining NAD^+^ induces a pseudohypoxic state disrupting nuclear mitochondrial communication during aging. *Cell*.

[13] Mouchiroud L., Houtkooper R. H., Moullan N. (2013). The NAD^+^/sirtuin pathway modulates longevity through activation of mitochondrial UPR and FOXO signaling. *Cell*.

[14] Rimmelé P., Bigarella C. L., Liang R. (2014). Aging-like phenotype and defective lineage specification in SIRT1-deleted hematopoietic stem and progenitor cells. *Stem Cell Reports*.

[15] Beckman K. B., Ames B. N. (1998). The free radical theory of aging matures. *Physiological Reviews*.

[16] Harman D. (1972). Free radical theory of aging: dietary implications. *American Journal of Clinical Nutrition*.

[17] Dröge W. (2002). Aging-related changes in the thiol/disulfide redox state: implications for the use of thiol antioxidants. *Experimental Gerontology*.

[18] Takubo K., Nagamatsu G., Kobayashi C. I. (2013). Regulation of glycolysis by Pdk functions as a metabolic checkpoint for cell cycle quiescence in hematopoietic stem cells. *Cell Stem Cell*.

[19] Yu W.-M., Liu X., Shen J. (2013). Metabolic regulation by the mitochondrial phosphatase PTPMT1 is required for hematopoietic stem cell differentiation. *Cell Stem Cell*.

[20] Zhang J., Khvorostov I., Hong J. S. (2011). UCP2 regulates energy metabolism and differentiation potential of human pluripotent stem cells. *The EMBO Journal*.

[21] Dansen T. B., Smits L. M. M., van Triest M. H. (2009). Redox-sensitive cysteines bridge p300/CBP-mediated acetylation and FoxO4 activity. *Nature Chemical Biology*.

[22] Guo Z., Kozlov S., Lavin M. F., Person M. D., Paull T. T. (2010). ATM activation by oxidative stress. *Science*.

[23] Velu C. S., Niture S. K., Doneanu C. E., Pattabiraman N., Srivenugopal K. S. (2007). Human p53 is inhibited by glutathionylation of cysteines present in the proximal DNA-Binding domain during oxidative stress. *Biochemistry*.

[24] Anastasiou D., Poulogiannis G., Asara J. M. (2011). Inhibition of pyruvate kinase M2 by reactive oxygen species contributes to cellular antioxidant responses. *Science*.

[25] Brunelle J. K., Bell E. L., Quesada N. M. (2005). Pxygen sensing requires mitochondrial ROS but not oxidative phosphorylation. *Cell Metabolism*.

[26] Sarbassov D. D., Sabatini D. M. (2005). Redox regulation of the nutrient-sensitive raptor-mTOR pathway and complex. *The Journal of Biological Chemistry*.

[27] De Bock K., Georgiadou M., Schoors S. (2013). Role of PFKFB3-driven glycolysis in vessel sprouting. *Cell*.

[28] Gut P., Verdin E. (2013). The nexus of chromatin regulation and intermediary metabolism. *Nature*.

[29] Lew C. R., Tolan D. R. (2013). Aldolase sequesters WASP and affects WASP/Arp2/3-stimulated actin dynamics. *Journal of Cellular Biochemistry*.

[30] Sutendra G., Kinnaird A., Dromparis P. (2014). A nuclear pyruvate dehydrogenase complex is important for the generation of acetyl-CoA and histone acetylation. *Cell*.

[31] Yang W., Xia Y., Ji H. (2011). Nuclear PKM2 regulates *β*-catenin transactivation upon EGFR activation. *Nature*.

[32] Dai Y., Grant S. (2010). New insights into checkpoint kinase 1 in the DNA damage response signaling network. *Clinical Cancer Research*.

[33] Anuranjani, Bala M. (2014). Concerted action of Nrf2-ARE pathway, MRN complex, HMGB1 and inflammatory cytokines—implication in modification of radiation damage. *Redox Biology*.

[34] Stagni V., Oropallo V., Fianco G., Antonelli M., Cinà I., Barilà D. (2014). Tug of war between survival and death: exploring ATM function in cancer. *International Journal of Molecular Sciences*.

[35] Stagni V., Santini S., Barilà D. (2014). ITCH E3 ligase in ATM network. *Oncoscience*.

[36] Allen D. M., van Praag H., Ray J. (2001). Ataxia telangiectasia mutated is essential during adult neurogenesis. *Genes and Development*.

[37] Kim J., Wong P. K. Y. (2009). Loss of ATM impairs proliferation of neural stem cells through oxidative stress-mediated p38 MAPK signaling. *STEM CELLS*.

[38] Kim J., Hwangbo J., Wong P. K. Y. (2011). P38 mapk-mediated bmi-1 down-regulation and defective proliferation in atm-deficient neural stem cells can be restored by akt activation. *PLoS ONE*.

[39] Carlessi L., De Filippis L., Lecis D., Vescovi A., Delia D. (2009). DNA-damage response, survival and differentiation in vitro of a human neural stem cell line in relation to ATM expression. *Cell Death and Differentiation*.

[40] Bernassola F., Karin M., Ciechanover A., Melino G. (2008). The HECT family of E3 ubiquitin ligases: multiple players in cancer development. *Cancer Cell*.

[41] van Schooneveld E., Wildiers H., Vergote I., Vermeulen P. B., Dirix L. Y., Van Laere S. J. (2015). Dysregulation of micro RNAs in breasts cancer and their potential role as prognostic and predictive biomarkers in patient management. *Breast Cancer Research*.

[42] Yan D., Ng W. L., Zhang X. (2010). Targeting DNA-PKcs and ATM with miR-101 sensitizes tumors to radiation. *PLoS ONE*.

[43] Qu C., Liang Z., Huang J. (2012). MiR-205 determines the radioresistance of human nasopharyngeal carcinoma by directly targeting PTEN. *Cell Cycle*.

[44] Grosso S., Doyen J., Parks S. K. (2013). MiR-210 promotes a hypoxic phenotype and increases radioresistance in human lung cancer cell lines. *Cell Death & Disease*.

[45] Svoboda M., Sana J., Fabian P. (2012). MicroRNA expression profile associated with response to neoadjuvant chemoradiotherapy in locally advanced rectal cancer patients. *Radiation Oncology*.

[46] Mognato M., Celotti L. (2015). MicroRNAs used in combination with anti-cancer treatments can enhance therapy efficacy. *Mini-Reviews in Medicinal Chemistry*.

[47] Hall E. J. (2007). Cancer caused by x-rays—a random event?. *The Lancet Oncology*.

[48] Begg A. C., Stewart F. A., Vens C. (2011). Strategies to improve radiotherapy with targeted drugs. *Nature Reviews Cancer*.

[49] Baskar R. (2010). Emerging role of radiation induced bystander effects: cell communications and carcinogenesis. *Genome Integrity*.

[50] Blyth B. J., Sykes P. J. (2011). Radiation-induced bystander effects: what are they, and how relevant are they to human radiation exposures?. *Radiation Research*.

[51] Panganiban R.-A. M., Snow A. L., Day R. M. (2013). Mechanisms of radiation toxicity in transformed and non-transformed cells. *International Journal of Molecular Sciences*.

[52] Najafi M., Fardid R., Hadadi G., Fardid M. (2014). The mechanisms of radiation-induced bystander effect. *Journal of Biomedical Physics and Engineering*.

[53] Coppe J.-P., Desprez P.-Y., Krtolica A., Campisi J. (2010). The senescence-associated secretory phenotype: the dark side of tumor suppression. *Annual Review of Pathology: Mechanisms of Disease*.

[54] Davalos A. R., Coppe J.-P., Campisi J., Desprez P.-Y. (2010). Senescent cells as a source of inflammatory factors for tumor progression. *Cancer and Metastasis Reviews*.

[55] Kumazaki T., Robetorye R. S., Robetorye S. C., Smith J. R. (1991). Fibronectin expression increases during in vitro cellular senescence: correlation with increased cell area. *Experimental Cell Research*.

[56] Sabin R. J., Anderson R. M. (2011). Cellular Senescence-its role in cancer and the response to ionizing radiation. *Genome Integrity*.

[57] Mladenov E., Magin S., Soni A., Iliakis G. (2013). DNA double-strand break repair as determinant of cellular radiosensitivity to killing and target in radiation therapy. *Frontiers in Oncology*.

[58] Di Maggio F. M., Minafra L., Forte G. I. (2015). Portrait of inflammatory response to ionizing radiation treatment. *Journal of Inflammation*.

[59] Meng Y., Efimova E. V., Hamzeh K. W. (2012). Radiation-inducible immunotherapy for cancer: senescent tumor cells as a cancer vaccine. *Molecular Therapy*.

[60] Meng Y. R., Beckett M. A., Liang H. (2010). Blockade of tumor necrosis factor *α* signaling in tumor-associated macrophages as a radiosensitizing strategy. *Cancer Research*.

[61] Formenti S. C., Demaria S. (2013). Combining radiotherapy and cancer immunotherapy: a paradigm shift. *Journal of the National Cancer Institute*.

